# Global climate change and above- belowground insect herbivore interactions

**DOI:** 10.3389/fpls.2013.00412

**Published:** 2013-10-22

**Authors:** Scott W. McKenzie, William T. Hentley, Rosemary S. Hails, T. Hefin Jones, Adam J. Vanbergen, Scott N. Johnson

**Affiliations:** ^1^Centre for Ecology and HydrologyWallingford, Oxfordshire, UK; ^2^The James Hutton InstituteDundee, UK; ^3^Centre for Ecology and HydrologyPenicuik, Midlothian, UK; ^4^Cardiff School of Biosciences, Cardiff UniversityCardiff, UK; ^5^Hawkesbury Institute for the Environment, University of Western SydneySydney, NSW, Australia

**Keywords:** root, soil biota, trophic interactions, CO_2_, climate change, folivory

## Abstract

Predicted changes to the Earth’s climate are likely to affect above–belowground interactions. Our understanding is limited, however, by past focus on two-species aboveground interactions mostly ignoring belowground influences. Despite their importance to ecosystem processes, there remains a dearth of empirical evidence showing how climate change will affect above–belowground interactions. The responses of above- and belowground organisms to climate change are likely to differ given the fundamentally different niches they inhabit. Yet there are few studies that address the biological and ecological reactions of belowground herbivores to environmental conditions in current and future climates. Even fewer studies investigate the consequences of climate change for above–belowground interactions between herbivores and other organisms; those that do provide no evidence of a directed response. This paper highlights the importance of considering the belowground fauna when making predictions on the effects of climate change on plant-mediated interspecific interactions.

## INTRODUCTION

Trophic interactions are likely to be crucial in shaping net effects of global climate change on ecosystems (e.g., Harrington et al., 1999; Tylianakis et al., 2008). Modified interactions between trophic groups (e.g., spatial or phenological decoupling of herbivore and predator populations) could have far reaching consequences across a range of natural and managed ecosystems with implications for food security (Gregory et al., 2009). In particular, the plant-mediated interactions between above- and belowground herbivores (Gange and Brown, 1989; Blossey and Hunt-Joshi, 2003; Johnson et al., 2012) may be important in the structuring of herbivore and multi-trophic communities (Bardgett and Wardle, 2010; Megías and Müller, 2010; Soler et al., 2012; Johnson et al., 2013). Surprisingly, investigating the potential impacts of climate change on above–belowground interactions, has received little attention (Schroter et al., 2004). Given that root and shoot herbivores affect plants in dramatically different ways, but also interact with each other (Meyer et al., 2009), the conclusions drawn from studies of climate change impacts limited to only aboveground herbivores may be misleading.

This perspectives paper uses empirical examples to illustrate how belowground herbivores influence aboveground plant–insect interactions. It draws on studies concerning above–belowground interactions as well as studies showing how climate change can alter soil herbivore communities. Finally, it considers the few examples that exist where above–belowground interactions have been studied under climate change scenarios to show how such plant-mediated interactions are, or may be, modified. Thus, this paper will highlight the potential for incomplete or inaccurate predictions of climate change impacts on plant–insect relationships, because of lack of consideration of belowground interactions.

## ABOVE–BELOWGROUND INTERACTIONS IN THE CURRENT CLIMATE

Studies of plant-mediated interactions between spatially separated herbivores have revealed contrasting ecological patterns (van Dam and Heil, 2011) that have evolved and built upon two major hypotheses: the Stress Response Hypothesis (Masters et al., 1993; Bezemer et al., 2004) and the Defense Induction Hypothesis (Bezemer et al., 2002). The Stress Response Hypothesis suggests root herbivory impairs the plant’s capacity for water and nutrient uptake, which can lead to the accumulation of nitrogen compounds in foliage (White, 1984) to increase palatability to aboveground herbivores. In contrast, the Defense Induction Hypothesis, suggests that belowground herbivores will induce a systemic increase in plant-defense chemicals, making it more difficult for herbivore colonization to occur aboveground (Bezemer and van Dam, 2005; Kaplan et al., 2008). These plant-mediated mechanisms arise through a complex path of communication between root and shoot tissues involving primary (e.g., Johnson et al., 2009) and secondary (Bezemer and van Dam, 2005) chemicals. The nature and mode of signaling between roots and leaves is a rapidly expanding area of research (Rasmann and Agrawal, 2008). Some hypotheses suggest that interactions between phytohormonal pathways regulate interspecific herbivore interactions (Soler et al., 2013). Different feeding guilds elicit different phytohormonal pathways. For example, jasmonic acid (induced by root-chewers) reduces a plant’s salicylic acid defense response against aphids (Soler et al., 2013). Given that above- and belowground herbivores can systemically alter the defensive phenotype of plants, future models of plant defense allocation would benefit greatly from a systemic-plant approach (Rasmann et al., 2009).

The consequences of interactions between spatially segregated organisms are more far-reaching than simple pair-wise herbivore–herbivore interactions, with effects cascading across species networks spanning trophic levels and the above- and belowground sub-systems (Scheu, 2001; Bardgett and Wardle, 2003; Wardle et al., 2004). The effects of root herbivory can, for instance, affect tertiary trophic levels. Root herbivores such as the cabbage root fly (*Delia radicum*) have been observed to affect, via the host plant, an aboveground herbivore (*Pieris brassicae*), its parasitoid (*Cotesia glomerata*), and hyper-parasitoid (*Lysibia nana*) (Soler et al., 2005). In this instance, *D. radicum* increased the development time of *P. brassicae* and *C. glomerata*, and the body size of both parasitoid and hyper-parasitoid were reduced. These effects were attributed to an alteration in the blend of phytotoxins (glucosinolates) emitted post-herbivory (Soler et al., 2005). Conversely, aboveground herbivory can have a negative effect on belowground herbivores and associated natural enemies (Jones and Finch, 1987; Soler et al., 2007). For instance, the presence of butterfly larvae (*P. brassicae*) reduced the abundance of the belowground herbivore (*D. radicum*) and its parasitoid (*Trybliographa rapae*) by up to 50% and decreased the body size of emerging parasitoid and root herbivore adults (Soler et al., 2007). If these broader interactions between organisms inhabiting the plant rhizosphere and canopy are typical, they could scale-up to play important roles in governing ecosystem function.

## CLIMATE CHANGE AND BELOWGROUND HERBIVORES

Many studies and comprehensive reviews address the effects of global climate change on aboveground insect herbivores (e.g., Bale et al., 2002; Cornelissen, 2011), whereas there are substantially fewer studies of the impacts on belowground organisms (Staley and Johnson, 2008). Soil fauna are, at least to some extent, buffered from the direct impacts of climate change (Bale et al., 2002). Carbon dioxide concentrations are already high within the soil due to root respiration and microbial processes (Haimi et al., 2005), and therefore soil fauna are less likely to be affected by increased atmospheric CO_2_ directly. Soil fauna may, however, be affected indirectly by increased growth of root resources caused by increased atmospheric CO_2_ (Norby, 1994). While higher soil temperature may also increase root growth, temperature increase may directly affect soil herbivore development and insect phenology (van Asch et al., 2007). Reduced soil moisture, potentially a consequence of increased temperature, can also impact many soil insect life-history traits, such as survival and abundance (Pacchioli and Hower, 2004). Predicted increases in climatic extremes under a future climate (e.g., increased flooding and drought events) may also drown or desiccate soil biota and herbivores, thus reducing their prevalence in the soil (Parmesan et al., 2000).

Soil-dwelling insect herbivores feed on the roots and therefore have very different effects on plant traits than their aboveground counterparts. These effects may alter the predicted consequences of global climate change on shoot herbivores (Robinson et al., 2012; Zavala et al., 2013). For instance, most plants increase biomass accumulation and rates of photosynthesis in response to elevated CO_2_ (Ainsworth and Long, 2005); this depends on plants maximizing water and nitrogen use efficiency. To facilitate this, many plants increase their root:shoot biomass ratio in response to elevated CO_2_, but this may be compromised by root herbivores, which remove root mass, therefore impairing water and nutrient uptake (Johnson and Murray, 2008). A recent meta-analysis by Zvereva and Kozlov (2012) showed that root herbivores reduced rates of photosynthesis in host plants; this contrasts with many aboveground herbivores that actually stimulate it (e.g., Thomson et al., 2003). Empirical evidence also suggests that root herbivory can effectively reverse the effects of elevated CO_2_ on eucalypt chemistry (e.g., increased foliar C:N ratio) and biomass, potentially altering the outcomes for aboveground herbivores (Johnson and Riegler, 2013).

## CLIMATE CHANGE AND ABOVE–BELOWGROUND INTERACTIONS: EMPIRICAL EVIDENCE

To our knowledge, there are only two peer-reviewed published examples describing how an elevated CO_2_ environment affects the interaction between above- and belowground herbivores. The first focused on the interaction between the root-feeding (*Pemphigus populitransversus*) and shoot-feeding (*Aphis fabae fabae*) aphids, on *Cardamine pratensis* (Salt et al., 1996). The study concluded the interaction between these spatially separated aphids was unaffected by CO_2_, because root herbivore populations were always smaller in the presence of an aboveground herbivore regardless of the CO_2_ environment. The second study investigated the conspecific interaction between aboveground adults and belowground larvae of the clover root weevil (*Sitona lepidus*) (Johnson and McNicol, 2010). Elevated CO_2_ increased leaf consumption by adult weevils but resulted in lower rates of oviposition. These patterns were interpreted by the authors to be a compensatory feeding response to reduced leaf nitrogen and lower reproductive output due to inadequate nutrition. Despite reduced rates of oviposition, larval survival was much greater at elevated than at ambient CO_2_-levels potentially due to increased nodulation (increased food source) of the host plant (*Trifolium repens*) under elevated CO_2_ conditions (Johnson and McNicol, 2010).

Enrichment with CO_2_ is not only expected to increase plant biomass both above- and belowground, but also to reduce plant tissue quality through increases in the C:N ratio and secondary metabolite concentrations (Bezemer and Jones, 1998). Compensatory feeding by phytophagous insects in an elevated CO_2_ environment may thus increase exposure to defensive chemicals present in plant tissue. This is likely, however, to be contingent on plant taxonomic identity, as concentrations of defensive chemicals may increase [e.g., glucosinolates in *Aradopsis thaliana* (Bidart-Bouzat et al., 2005)], or remain unchanged [e.g., tannins in *Quercus myrtifolia* (Rossi et al., 2004)] in response to CO_2_ enrichment.

Temperature changes may alter above–belowground interactions either by affecting invertebrate phenology directly (Gordo and Sanz, 2005; Harrington et al., 2007) or indirectly through changes in the plant (Harrington et al., 1999; Bale et al., 2002; Singer and Parmesan, 2010), although this remains to be tested empirically. A predicted increase in global mean temperatures may also result in an increased water stress response in plants (Huberty and Denno, 2004), making them more susceptible to herbivory both above- and belowground.

Summer drought is another factor associated with climate change that has been shown to influence above–belowground interactions. Typically, root-chewing *Agriotes* sp. larvae reduced the abundance and performance of leaf-mining *Stephensia brunnichella* larvae and its associated parasitoid (Staley et al., 2007). This effect was, however, negated under drought conditions. Changes to summer rainfall may, therefore, reduce the occurrence or alter the outcome of plant-mediated interactions between insect herbivores.

Above–belowground interactions may also be influenced by variation in soil moisture. Experimentally elevated rainfall increased the suppression of an outbreak of the herbivorous moth larvae *Hepialus californicus* by an entomopathogenic nematode (*Heterorhabditis marelatus*), thereby indirectly protecting the host plant – bush lupine (*Lupinus arboreus*) (Preisser and Strong, 2004). Thus climate change, by altering patterns of precipitation, has the potential to modify herbivore–natural enemy interactions to reduce herbivore pressure.

Few studies have integrated the multiple abiotic factors associated with climate change (i.e., water supply, temperature, CO_2_, etc.) to investigate their combined effects on above–belowground interactions. One such study (Stevnbak et al., 2012) manipulated CO_2_ concentration, air and soil temperature, and precipitation to show that soil microbial biomass was altered by aboveground herbivory (*Chorthippus brunneus*). The combination of multiple climate change treatments with aboveground herbivory increased microbivorous protist abundance in the soil, emphasizing the importance of considering climate change in above–belowground interactions.

## THE FUTURE OF ABOVE–BELOWGROUND INTERACTIONS AND CLIMATE CHANGE RESEARCH

Johnson et al. (2012) conducted a meta-analysis on two-species above–belowground herbivore interactions. Although restricted by not including other trophic groups, the meta-analysis did identify several factors that determine the outcomes of interactions between spatially separated herbivores. From these outcomes it is possible to develop hypotheses of how specific interactions are likely to be affected by climate change. The chronological sequence in which herbivores fed on shared plants was a major determinant of interaction outcome. In particular, aboveground herbivores negatively affected belowground herbivores when they fed first, but not when feeding synchronously or following belowground herbivores. Conversely, belowground herbivores typically had positive effects on aboveground herbivores only when synchronously feeding, otherwise they had a negative impact (Johnson et al., 2012). Many of the data on aboveground species are from aphids; we know that elevated CO_2_ and temperature results in earlier and longer seasonal occurrences of many pest species, including aphids (Harrington et al., 2007). Therefore in the future it might be reasonable to expect that some aphids may initiate feeding on the plant prior to belowground herbivores. Under such circumstances, aphids may negatively affect the belowground herbivore while remaining unaffected themselves, the reverse of the interaction under current conditions. Likewise, if drought conditions delayed root herbivore development this change could become even more pronounced.

Feeding guild identity (e.g., chewers, suckers, gallers) can affect the outcome of above–belowground interactions. Johnson et al. (2012) showed that the effects on aboveground herbivores depended on belowground herbivore guild. Individual feeding guilds and trophic levels respond differently to climate change (Voigt et al., 2003), but how this translates into changes in above–belowground trophic interactions remains unexplored. The increased level of defense compounds in plant tissue, predicted to occur under climate change scenarios (Robinson et al., 2012), are likely to have a disproportionate effect between (a) herbivores feeding above- or belowground: defense compounds may be concentrated in either leaf or root tissue, and (b) different feeding guilds: chewing insects being more susceptible to defensive compounds than phloem-feeders. There is, however, a strong bias in the literature, with certain herbivore guilds and orders (e.g., Lepidoptera) having been represented disproportionately within empirical studies (Robinson et al., 2012). Conclusions extrapolated regarding general herbivore-responses to climate change should, therefore, be treated with appropriate caution.

There are few long-term above–belowground interaction studies. Some Arctic long-term manipulative field studies (e.g., Ruess et al., 1999) that illustrate the effects of climate warming on soil fauna provide essential information on legacy effects in natural ecosystems. These indicate that above–belowground interactions may be separated temporally (Kostenko et al., 2012) as well as spatially. Long-term field experiments may also yield different results to laboratory experiments conducted over a smaller timescale (Johnson et al., 2012).

## CONCLUSION AND RESEARCH AGENDA

Our understanding of how individual species respond to climate change has increased dramatically over the past 25 years. We have a relatively well-informed understanding of how aboveground herbivores may react to different aspects of climate change (e.g., Bale et al., 2002) but our knowledge of belowground species responses remains lacking. Johnson and Murray (2008) illustrate how this area of research is a “hot topic” for multidisciplinary research while others (Soler et al., 2005; van Dam and Heil, 2011) underline the importance of a more integrated understanding of climate change impacts on ecosystems that incorporates above- and belowground trophic linkages.

Based on current knowledge of above–belowground interactions we are able to formulate hypotheses that could be tested empirically in future research. For example:

(1) Root herbivory is likely to change fundamentally plant responses to an elevated CO_2_ environment, since root function usually underpins the plants ability to respond to environmental changes. We hypothesise that inclusion of root herbivores will reverse the effects of elevated CO_2_ on certain aboveground herbivores, particularly those negatively affected by higher C:N ratios (e.g., leaf-miners).
(2) Plant functional identity may shape how above–belowground interactions respond to climate change. For instance, plants with C_3_ and C_4_ photosynthetic pathways will respond differently to climate change, and notably elevated CO_2_ (Barbehenn et al., 2004a). In particular, C_3_ plants potentially show a greater decline in nutritional quality than C_4_ plants, which are often inherently less favorable hosts to insect herbivores (see the C_3_–C_4_ hypothesis of Caswell et al., 1973). This might lead to compensatory feeding on C_3_, but not C_4_, plants in future climates (Barbehenn et al., 2004b). We hypothesis that above–belowground interactions are likely to be more affected on C_3_ than C_4_ plants.
(3) Belowground herbivory induces a water stress on the plant, similar to drought. Experiments investigating drought effects on aboveground plant-herbivore interactions may, therefore, be analogous to above–belowground herbivore interactions generally. We hypothesise that the combination of a drought treatment and a belowground herbivore may have additive negative effects on the plant and consequently on aboveground herbivores (through increased susceptibility to herbivory).

Increasing trophic complexity in empirical climate change research will strengthen the ability to make more accurate predictions of trophic interactions in future environments (Robinson et al., 2012). Making predictions based on simple plant–herbivore interactions compared to wider communities may be misleading and interaction outcomes may be altered with the inclusion of higher trophic levels. As seen aboveground, climate change may not directly affect the abundance of a herbivore, however, if the abundance or impact of an associated antagonist is reduced then climate change may increase herbivore abundance indirectly. Disrupted phenological synchrony between predator and prey (Hance et al., 2007) may be one mechanism, another may be a reduction in plant production of chemical attractants (synomones) that recruit natural enemies, which then regulate herbivore numbers (Yuan et al., 2009). Alternatively, climate change may benefit the prey and antagonist equally, with any increase in herbivore abundance merely supporting greater numbers of natural enemies and thus leading to no net change in populations (e.g., Chen et al., 2005). An integrated approach considering trophic interactions as an integral part of an ecosystem comprising above- and belowground components will provide a more accurate estimation of climate change impacts. For example, a positive effect of root herbivores on folivores at higher temperatures may, if climate change positively affected antagonist efficacy (e.g., Bezemer et al., 1998; Hance et al., 2007), be canceled-out with the inclusion of an above- or belowground antagonist. For the most part this remains to be tested empirically. Moreover, with more empirical data it may be possible that – as has been observed with other areas of climate change research (Robinson et al., 2012) – apparent idiosyncratic outcomes of climate change impacts on plant-herbivore interactions give way to reveal generalities. Trends have become apparent in some aspects of insect herbivory in elevated CO_2_ (Zavala et al., 2013), for example, phloem feeders generally increase in abundance under elevated CO_2_, whereas leaf-miners generally decrease (Robinson et al., 2012). Alternatively, further research may simply reveal a lack of general responses of above–belowground interactions to climate change. For instance, despite the large body of research on aphid–plant interactions under climate change, aphid responses to CO_2_ enrichment still appear to be highly species-specific (see Sun and Ge, 2011 and references therein). The challenge for ecologists therefore is to utilize current knowledge of individual species responses to climate change and develop our understanding into general hypotheses for functional guilds, networks of species and ecosystem processes.

## Conflict of Interest Statement

The authors declare that the research was conducted in the absence of any commercial or financial relationships that could be construed as a potential conflict of interest.
